# Engraftment potential of maternal adipose-derived stem cells for fetal transplantation

**DOI:** 10.1016/j.heliyon.2020.e03409

**Published:** 2020-03-04

**Authors:** Akihiro Kawashima, Rika Yasuhara, Ryosuke Akino, Kenji Mishima, Michiko Nasu, Akihiko Sekizawa

**Affiliations:** a Department of Obstetrics and Gynecology, Showa University School of Medicine, 1-5-8 Hatanodai, Shinagawa, Tokyo, 142-8666, Japan; b Division of Pathology, Department of Oral Diagnostic Sciences, Showa University School of Dentistry, 1-5-8 Hatanodai, Shinagawa, Tokyo, 142-8555, Japan

**Keywords:** Neuroscience, Nervous system, Cell differentiation, Stem cells research, Tissue culture, Regenerative medicine, Neurogenesis, Mesenchymal stem cell, In utero transplantation, Adipose tissue-derived stem cell

## Abstract

Advances in prenatal molecular testing have made it possible to diagnose most genetic disorders early in gestation. In utero mesenchymal stem cell (MSC) therapy can be a powerful tool to cure the incurable. With this in mind, this method could ameliorate potential physical and functional damage. However, the presence of maternal T cells trafficking in the fetus during pregnancy is thought to be the major barrier to achieving the engraftment into the fetus. We investigated the possibility of using maternal adipose-derived stem cells (ADSCs) for in utero transplantation to improve engraftment, thus lowering the risk of graft rejection. Herein, fetal brain engraftment using congenic and maternal ADSC grafts was examined via in utero stem cell transplantation in a mouse model. ADSCs were purified using the mesenchymal stem cell markers, PDGFRα, and Sca-1 via fluorescence-activated cell sorting. The PDGFRα^+^Sca-1^+^ ADSCs were transplanted into the fetal intracerebroventricular (ICV) at E14.5. The transplanted grafts grew for at least 28 days after in utero transplantation with PDGFRα^+^Sca-1^+^ ADSC, and mature neuronal markers were also detected in the grafts. Furthermore, using the maternal sorted ADSCs suppressed the innate immune response, preventing the infiltration of CD8 T cells into the graft. Thus, in utero transplantation into the fetal ICV with the maternal PDGFRα^+^Sca-1^+^ ADSCs may be beneficial for the treatment of congenital neurological diseases because of the ability to reduce the responses after in utero stem cell transplantation and differentiate into neuronal lineages.

## 1 Introduction

Neurometabolic disorders in children differ from those in adults in that most are autosomal recessively inherited, and few have X-linked inheritance [1]. These disorders may be catabolic and anabolic conditions, some of which combine maldevelopment and degenerative features [2]. Many congenital genetic diseases can now be diagnosed early in gestation of the fetus using fetal cells or cell-free fetal DNA present in the maternal blood [3, 4]. However, there are few prenatal treatments available. Stem cell-based therapy using mesenchymal stem cells (MSCs) is being explored in a large number of clinical trials, including for possible treatment of brain pathologies [5, 6]. Preemptive treatment of the fetus would completely transform the paradigm for treating genetic disorders [7]. When considering how to treat neurometabolic disorders in infants with effective results, in utero transplantation using MSCs is deployed to treat symptoms. This can prevent disease onset and avoid the devastating manifestations that would otherwise occur before birth. Moreover, in utero transplantation has also made it possible to safely deliver stem cells to precise anatomic sites within early gestation [8, 9].

Stereotactic intracranial administration of stem cells is proven safe in current human neurosurgical procedures [10, 11]. According to recent studies, the intracerebroventricular (ICV) administrated MSCs have been reported to activate neurogenesis in the subventricular zone (SVZ) and dentate gyrus where neural stem cells are maintained after embryonic development for the production of new cells in the brain [12]. One completed open-label Phase I trial shows that ICV transplantation of MSCs in the premature infant with severe intraventricular hemorrhage is safe and feasible [13]. One of the optimal routes for in utero stem cell transplantation into the fetal brain may be the ICV route, which can effectively deliver more MSCs to the SVZ where neurogenesis occurs.

The conventional method for isolation of MSCs is based on their adhesive nature as to plastic dishes [14]. MSCs prepared by this protocol are heterozygous and possibly include other adherent cells [15]. Mabuchi et al. established a means of flow cytometric isolation of MSCs using cell-surface MSC-specific markers, platelet-derived growth factor receptor alpha (PDGFRα), and stem cell antigen-1 (Sca-1), which enables us to obtain highly purified MSCs [16]. Adipose-derived stem cells (ADSCs) are a type of MSCs with the ability to differentiate into mesodermal lineages and ectodermal lineages as neuron-like cells and easily obtained in large quantities with little donor site morbidity or patient discomfort [17]. In addition, ADSCs can be modulated by other cells to start their differentiation process [18] and also have more significant neuronal potential than bone marrow MSCs (BM-MSCs) in vitro [19]. PDGFRα^+^Sca-1^+^ ADSCs can be an effective cell source to be used in neurodegenerative disorders.

MSCs were immunologically privileged and were subsequently promoted as safe to use in allogeneic settings without concern for immune rejection [20]. Emerging evidence shows that allogeneic MSCs can indeed induce a strong immune response in vivo, which may have severe consequences depending on the disease indication for which the cells are being administered [21]. Allogenic MSC transplantation leads to inflammation and to the initiation of effective mechanisms that coordinate innate and adaptive immune responses [22]. Efforts have been made to improve MSC immunosuppressive and tolerogenic potentials and to prolong MSC engraftment [23]. Previously, a fetus was considered immunologically privileged so that in utero transplantation was based on the introduction of donor cells into a fetus at an early stage of development resulting in the development of chimerism with a low risk of rejection of the donor cells because of the undeveloped fetal immune system [24]. However, the maternal immune response inhibits allogeneic stem cell engraftment as shown through multiple animal studies [25]. Successful in utero MSC transplantation might be more readily achievable with the mother as a donor. Taking advantage of pre-established fetal immune tolerance through very early natural acquisition might permit efficient delivery of a more massive cell dose. When using maternal stem cells for in utero transplantation, there is a problem with regard to passing the pathogenic copies from the mother. However, if the fetus is affected with the recessive diseases that occur due to damage in both copies, and the mother is a heterozygous carrier or a healthy individual, the maternal stem cells have copies of the healthy genes that the fetus needs. One recent study is a single center phase I clinical trial combining maternal bone marrow stem cell transplantation with intrauterine blood transfusion in alpha-thalassemia major, led by MacKenzie, University of California, San Francisco (www.fetaltherapies.org).

Herein, we investigated that PDGFRα^+^Sca-1^+^ ADSCs would have the ability to differentiate into neuronal lineages after in utero transplantation in a mouse model. We first examined whether PDGFRα^+^Sca-1^+^ ADSCs can differentiate into neural lineages in vitro using a three-dimensional culture. After we had proof of the neuronal capacity in PDGFRα^+^Sca-1^+^ ADSCs, we next investigated whether the PDGFRα^+^Sca-1^+^ ADSCs can differentiate into neuronal lineages after in utero transplantation via the fetal lateral ventricle. Following the investigation for the possibility of neural differentiation in PDGFRα^+^Sca-1^+^ ADSCs transplantation in vivo, we investigated whether PDGFRα^+^Sca-1^+^ ADSCs obtained from the mother contributes to a reduced or weakened the inflammation in the fetus after in utero transplantation.

## 2 Materials and methods

### 2.1 Isolation and culture of ADSCs

C57BL/6JJmsSlc (C57BL6/J) mice were provided by Sankyo Labo-service Corp (Tokyo, Japan). C57BL/6-TgN(CAG-EGFP) mice (congenic green mice) were acquired from RIKEN BRC (RBRC00267). To isolate ADSCs, we harvested inguinal fat tissue from 6–8-week-old female C57BL/6J and congenic green mice. Significant fasciae and blood vessels were detached under a dissecting microscope and trimmed into small pieces under sterile conditions. Then, the minced tissues were enzymatically digested with 1 mg/ml collagenase type I (Sigma-Aldrich, St. Louis, MO) in MEMα (Sigma-Aldrich, St. Louis, MO) for 90 min at 37 °C. The enzymes were inactivated with an equal volume of minimum essential medium-α (MEM-α; Thermo Fisher Scientific, Waltham, MA) supplemented with 10% fetal bovine serum (FBS; Thermo Fisher Scientific, Waltham, MA), and the samples were filtered through a 70-μm mesh filter to remove debris. The suspension was centrifuged at 300 ×g for 5 min to obtain cellular pellets. The cellular pellets were resuspended in the maintenance medium; MEM-α supplemented with 10% FBS, 1% penicillin/streptomycin solution (P/S; 100 IU/mL penicillin, 100 IU/mL streptomycin; Gibco, BRL, Palo Alto, CA). The resuspended cells were seeded at 22,000 cells/cm2 onto a culture dish and cultured in a humidified incubator at 37 °C with 5% carbon dioxide overnight. The floating cells were carefully removed via two washes with PBS, and then the maintenance medium was added for further culture, and the maintenance medium was changed every third day. The cultured cells were passaged at 80% confluency using TripLE Express (Thermo Fisher Scientific, Waltham, MA), and the dispersed cells were seeded at 22,000 cells/cm2 onto new dishes in the maintenance medium.

### 2.2 Purification of PDGFRα and Sca-1 positive ADSCs via flow cytometry

The third passaged attachment cells were used for the experiments. Overall, 1 × 10^6^ cells were suspended in 100 μl staining buffer (HBSS with 3% FBS) and were stained with APC-labeled anti-PDGFRα (BioLegend, San Diego, CA), PE/Cy7-labeled anti-Sca-1 (BioLegend, San Diego, CA), PE-labeled anti-mouse lineage cocktail (mCD3ε/Gr-1/CD11b/CD45R (B220)/Ter-119) (BioLegend, San Diego, CA), and PE-labeled anti-mouse CD31 (BioLegend, San Diego, CA) antibodies for 30 min on ice with dilutions as suggested by the manufacturer instructions. Lineage^−^CD31^−^PDGFRα^+^Sca-1^+^ cells were sorted using an SH800S cell sorter (SONY).

### 2.3 Induction of neural differentiation in ADSCs

We investigated neural differentiation potency of PDGFRα^+^Sca-1^+^ ADSCs. The sorted cells were formed into aggregates, and the aggregates were differentiated into neural lineages. PDGFRα^+^Sca-1^+^ ADSCs were harvested and seeded onto Nunclon Sphera 96U-well plate (Thermo Fisher Scientific, Waltham, MA) at a density of 10,000 cells in 100 μl MEMα supplemented with 20% KnockoutTM Serum Replacement (Thermo Fisher Scientific, Waltham, MA) per well. From days 0–6, the cells were treated with 100 ng/ml mouse Dickkopf-1 (R&D Systems, Minneapolis, MN) as an inhibitor of Wnt/β-catenin signaling, 10 μM SB431542 as an inhibitor of TGFβ signaling (Sigma-Aldrich, St. Louis, MO) and 0.1 mM 2-mercaptoethanol (Sigma-Aldrich, St. Louis, MO) to promote self-aggregation of the cells into spheroids. On day 9, cell aggregates were transferred into a non-adhesive dish (Sumilon PrimeSurface plate; Sumitomo Bakelite, Tokyo, Japan) and cultured in Neurobasal medium (Thermo Fisher Scientific, Waltham, MA) supplemented with 1% N2 supplement (Thermo Fisher Scientific, Waltham, MA), 1% B27 (Thermo Fisher Scientific, Waltham, MA), and 1% Glutamax (Thermo Fisher Scientific, Waltham, MA), and further cultured with 0.5 ng/ml recombinant mouse bone morphogenetic protein 4 (BMP4; R&D Systems, Minneapolis, MN) and 20 ng/ml Wnt 3a (R&D Systems, Minneapolis, MN) until day 11. On day 12, 20 ng/ml recombinant mouse basic fibroblast growth factor (bFGF; PeproTech EC, London, UK) and 20 ng/ml Epidermal Growth Factor (EGF; R&D Systems, Minneapolis, MN) were added to induce differentiation into neural lineages, because EGF and bFGF treatment enhances neuronal commitment from MSCs [26]. During days 13–21, cells were fed every other day with differentiation medium to induce differentiation.

### 2.4 In utero cell transplantation of PDGFRα^+^Sca-1^+^ ADSCs from green congenic mice

C57BL/6J pregnant mice on E14.5 were anesthetized with 2% isoflurane (Wako, Osaka, Japan) before the operation. Then the uterine horns were exposed. After that, 2.0 × 10^5^ cells PDGFRα^+^Sca-1^+^ ADSCs from green congenic mice in 2 μl of PBS with 1 μl of VitroGel 3D-RGD (TheWell Bioscience, NJ, USA) as a scaffold and 0.05% FastGreen FCF (Sigma-Aldrich, St. Louis, MO) to track the success of injection were injected into the lateral ventricle of each fetus through the uterine wall using a Hamilton® Neurosyringe (Hamilton, Reno, NV). The same volume of vehicle control (2 μl of PBS, 1 μl of VitroGel 3D-RGD and 0.05% FastGreen FCF) was injected into fetuses in C57BL/6J pregnant mice. The transplants were collected at 28 days post-transplantation and were examined immunohistochemically, to access whether transplanted PDGFRα^+^Sca-1^+^ ADSCs could differentiate into neuronal cells.

### 2.5 Lentiviral transfection into ADSCs and in utero cell transplantation of maternal ADSCs transfected with Zsgreen

Next, to evaluate the inflammation reactions after in utero transplantation with maternal PDGFRα^+^Sca-1^+^ ADSCs, we obtained PDGFRα^+^Sca-1^+^ ADSCs from each female C57BL/6J mouse at 6–8 weeks and mated the female mouse. To enable PDGFRα^+^Sca-1^+^ ADSC tracing after transplantation, PDGFRα^+^Sca-1^+^ ADSCs were transfected with ZsGreen (green fluorescent protein) gene via lentiviruses. Lentiviruses were prepared by transfecting pLVX-IRES-ZsGreen1 Vector (Takara, Shiga, Japan) into Lenti-X293T cells (Takara, Shiga, Japan) which facilitate optimal lentivirus production. Lentivirus particles were obtained according to the manufacture's instruction and were concentrated using a Lenti-X™ Concentrator (Takara, Shiga, Japan). 1.0 × 10^6^ cells ADSCs were infected with LV-ZsGreen and were cultured for 48 h. Next, ZsGreen-positive PDGFRα^+^Sca-1^+^ ADSCs were sorted using a cell sorter, as described above. The pregnant mouse at E14.5 was anesthetized with 2% isoflurane before the operation. 2.0 × 10^5^ cells Zsgreen-positive PDGFRα^+^Sca-1^+^ ADSCs from the pregnant mouse in 2 μl of PBS with 1 μl of VitroGel 3D-RGD and 0.05% FastGreen FCF were injected into the lateral ventricle of each fetus through the uterine wall using a Hamilton® Neurosyringe. To compare the inflammation reaction between maternal PDGFRα^+^Sca-1^+^ ADSCs and PDGFRα^+^Sca-1^+^ ADSCs from green congenic mice, we injected the PDGFRα^+^Sca-1^+^ ADSCs from act-EGFP mice into a lateral ventricle of the fetuses in C57BL/6J pregnant mice; 2.0 × 10^5^ cells ADSCs from act-EGFP mice in 2 μl of PBS with one μl of VitroGel 3D-RGD and 0.05% FastGreen FCF. The same volume of vehicle control (2 μl of PBS, 1 μl of VitroGel 3D-RGD, and 0.05% FastGreen FCF) was injected as a control into a lateral ventricle of the fetuses in C57BL/6J pregnant mice. The transplanted fetal brains were collected on 5 days after post-transplantation and examined by immunohistochemistry and RT-qPCR.

### 2.6 Immunofluorescence staining

The aggregates differentiated under neurogenic conditions for 9 days were washed with PBS and were fixed in 4% paraformaldehyde (Wako, Osaka, Japan) for 10 min at room temperature before freezing in OCT compound (Tissue Tek, Sakura Finetek, Torrance, CA). The transplanted brains were fixed in 4% paraformaldehyde overnight at 4 °C, and cryoprotection was performed using a sucrose gradient (up to 30% overnight). The brain tissues were sliced at 200 μm using Brain Matrices (0.5 mm) before freezing in OCT compound. Frozen sections (4 μm) of the aggregates and frozen sections (8 μm) of brain tissue were prepared using a cryostat (Thermo Fisher Scientific, Waltham, MA). The sliced tissues were fixed in 4% paraformaldehyde for 10 min, were washed with TBS, and were blocked using a serum-free blocking agent (Dako Denmark A/S, Glostrup, Denmark) for 1 h at room temperature in a humidity chamber. The cells were permeabilized using 0.1% Triton X-100 (Sigma-Aldrich, St. Louis, MO) in TBS before blocking. Cells were incubated with the following primary antibodies overnight at 4 °C: anti-SOX2 (mouse monoclonal/Cell Signaling Technology, Beverly, MA) at 1:100, anti-NESTIN (rabbit monoclonal/Abcam) at 1:100, anti-TUBB3 (rabbit monoclonal/Sigma-Aldrich, St. Louis, MO) at 1:100, anti-GFAP (mouse monoclonal/Millipore, Billerica, MA, USA) at 1:100, anti-CD45 (mouse monoclonal/BioLegend, San Diego, CA) at 1:100, and anti-CD8 (mouse monoclonal/Lifespan BioSciences, Seattle, WA) at 1:100. Then, sections were incubated with goat anti-rabbit Alexa Fluor-594 conjugated secondary antibodies (1: 200, Molecular Probes, Carlsbad, CA) for 1 h at room temperature, followed by nuclear labeling using 4′,6-diamidino-2-phenylindole dihydrochloride (DAPI, 50 μg/ml) for 10 min Z9000 fluorescence microscope (Keyence, Osaka, Japan) was used to observe and image fluorescent samples.

### 2.7 Gene expression analysis

We analyzed gene expression in the ADSC aggregates and the brain after transplantation. Total RNA was isolated using RNeasy Mini Kit (QIAGEN, Hilden, Germany). cDNA was generated from 2 μg of total RNA using SuperScript III reverse transcriptase ((Invitrogen, Carlsbad, CA). qPCR was performed using a 7500 Fast Real-Time PCR System (Applied Biosystems, Foster City, CA) using Fast SYBR Green Master Mix (Thermo Fisher Scientific, Waltham, MA) and a program comprising 40 cycles of 95 °C for 20 s, 60 °C for 3 s, and 60 °C for 20 s. The comparative quantification method (2−ΔΔCT) was used, and samples were normalized according to ACTB mRNA levels via SYBR green real-time PCR. Table 1 shows the primer sequences for each gene used in this study. All the samples, including the template controls, were assayed in duplicate.

**Table 1:** Sequences of oligonucleotide primers used for real-time PCR.

Gene	Forward primer sequence	Reverse primer sequence	Size of product (bp)
*Actb*	5′-AGAGGGAAATCGTGCGTGAC-3′	5′-CAATAGTGATGACCTGGCCGT-3′	138
*Gfap*	5′-TCCTGGAACAGCAAAACAAG-3′	5′-CAGCCTCAGGTTGGTTTCAT-3′	224
*Il10*	5′-ATAACTGCACCCACTTCCCA-3′	5′-GGGCATCACTTCTACCAGGT-3′	206
*Il1b*	5′-GCACTACAGGCTCCGAGATGAAC-3′	5′-TTGTCGTTGCTTGGTTCTCCTTGT-3′	147
*Nestin*	5′-CCAGAGCTGGACTGGAACTC-3′	5′-ACCTGCCTCTTTTGGTTCCT-3′	161
*Pdgfra*	5′-TATCCTCCCAAACGAGAATGAGA-3′	5′-GTGGTTGTAGTAGCAAGTGTACC-3′	226
*Sox2*	5′-GCGGAGTGGAAACTTTTGTCC-3′	5′-GGGAAGCGTGTACTTATCCTTCT-3′	156
*Tim1*	5′-ACATATCGTGGAATCACAACGAC-3′	5′-ACTGCTCTTCTGATAGGTGACA-3′	114
*Tnfa*	5′-CTGAACTTCGGGGTGATCGG-3′	5′-GGCTTGTCACTCGAATTTTGAGA-3′	122
*Tubb3*	5′-TAGACCCCAGCGGCAACTAT-3′	5′-GTTCCAGGTTCCAAGTCCACC-3′	127

### 2.8 Ethics

The study was conducted in compliance with the Declaration of Helsinki. The experimental protocols were explicitly approved by the Institutional Animal Care and Use Committee of Showa University School of Medicine (approval #08027).

### 2.9 Statistical analysis

Quantitative data are expressed as the mean ± SD. Statistical comparisons were performed by Student's *t*-test, one-way analysis of variance (ANOVA), and two-way ANOVA using GraphPad Prism 7 software (GraphPad Software, San Diego, CA). The Tukey post hoc test was used for all pairwise comparisons among groups. A probability of <0.05 was considered statistically significant.

## 3 Results

### 3.1 Neural cell differentiation of ADSC aggregates

**Figure 1:**
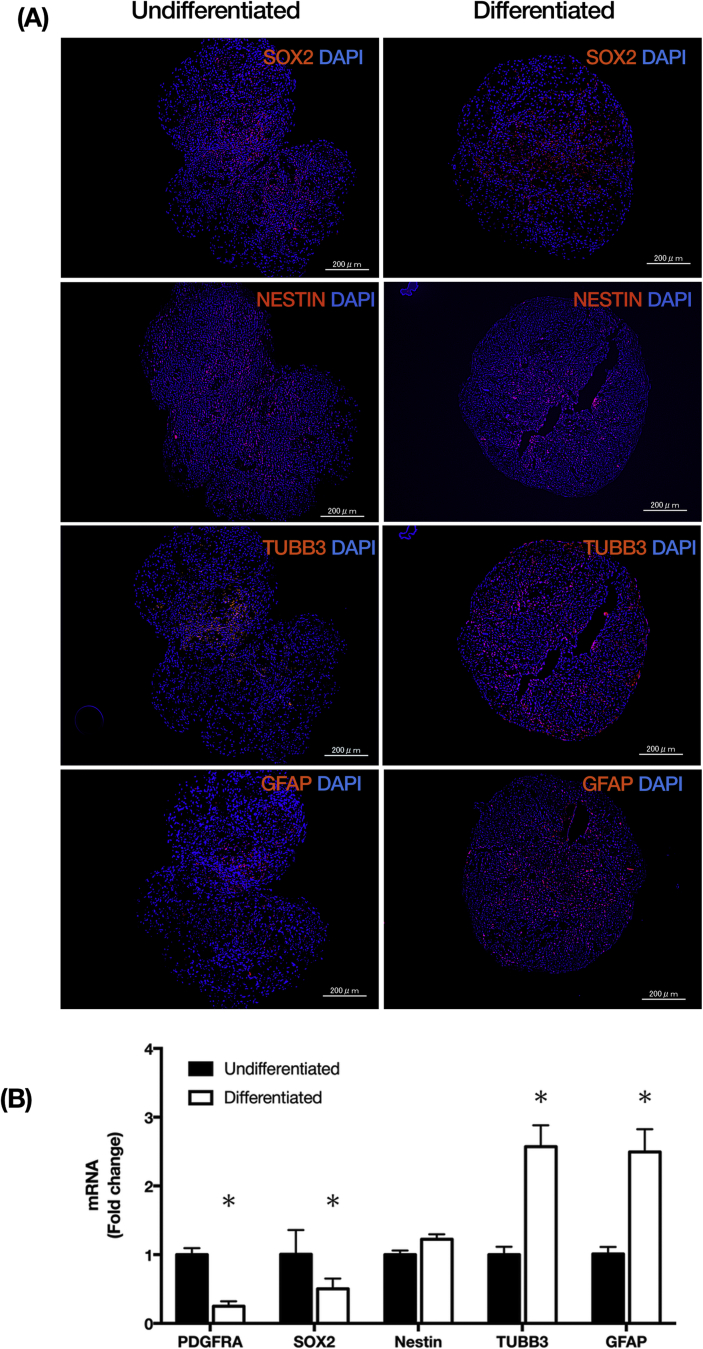
Characterization of PDGFRα^+^Sca-1^+^ ADSCs in suspension culture. Immuno-staining of PDGFRα^+^Sca-1^+^ ADSC aggregates on day 21 (differentiated PDGFRα^+^Sca-1^+^ ADSC) for the expression of neural lineage markers, SOX2 (A-a; red), NESTIN(A-b; red), TUBB3 (A-c; red) and GFAP (A-d; red) as compared to PDGFRα^+^Sca-1^+^ ADSC aggregates on day 12 (undifferentiated PDGFRα^+^Sca-1^+^ ADSCs). Nuclei were counterstained with DAPI (blue). Scales bar; 200 μm. (B): qRT-PCR data on the expression of a mesenchymal stem cell marker, *Pdgfra*, and marker neural lineage markers, *Sox2, Nestin, Tubb3*, and *Gfap* on day 12 (undifferentiated) and day 21 (differentiated) PDGFRα^+^Sca-1^+^ ADSC aggregates. All mRNA expression levels were normalized to the reference gene Actb expression, data presented here as fold increase of mean ± SD over PDGFRα^+^Sca-1^+^ ADSCs undifferentiated cells. Abbreviations: DAPI, 4′, 6-diamidoino-2-phenylindole; qRT-PCR, quantitative reverse transcription-polymerase chain reaction; Actb, Actin Beta.

Primary adipose-derived stem cells (ADSCs) were isolated from the inguinal fat pad of 6–8-week-old mice and cultured. Approximately 70% of the second passaged ADSCs were positive for the mesenchymal stem cell markers Sca-1 and PDGFRα and negative for lineage markers (data not shown). First, we examined whether PDGFRα^+^Sca-1^+^ ADSCs could differentiate into neural lineage cells using a method for inducing neural differentiation. On day 12, undifferentiated aggregates robustly expressed the neural stem cell makers SOX2 and NESTIN, but the slight expression of a neuron marker, TUBB3, and an astrocyte marker, GFAP, was detected (Figure 1A, undifferentiated). On day 21, expression of the neural stem cell markers was reduced in differentiated ADSC aggregates, and the expression of TUBB3 and GFAP was increased (Figure 1A, differentiated). We also examined the gene expression of 12- (undifferentiated) and 21-day cultured (differentiated) aggregates. The gene expressions of the MSC markers, *Pdgfra* and *Sox2*, were significantly reduced in the differentiated aggregates compared with that in undifferentiated aggregates; conversely, the gene expressions of *Tubb3* and *Gfap* were increased in the differentiated aggregates (Figure 1B). These results suggest that PDGFRα^+^Sca-1^+^ ADSCs might have the potential to differentiate into neural lineage cells in the aggregation culture.

### 3.2 PDGFRα^+^Sca-1^+^ ADSCs from green congenic mice in utero transplantation

**Figure 2:**
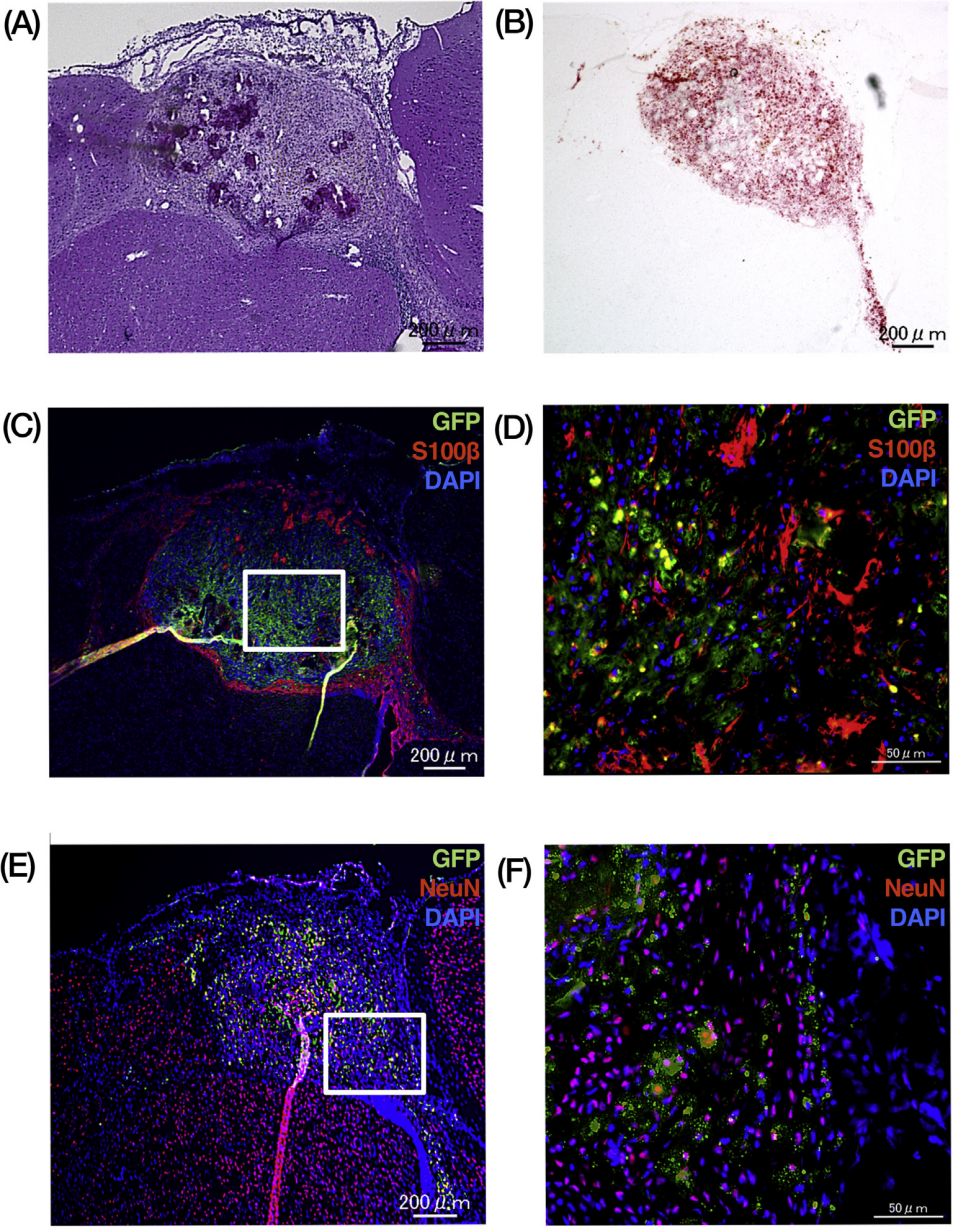
Engraftment of PDGFRα^+^Sca-1^+^ ADSCs from green congenic mice in the fetal brain. (A) The sections were stained using hematoxylin-eosin. (B) Oil red-O staining revealed the presence of intracellular lipid droplets (red) in the transplanted PDGFRα^+^Sca-1^+^ ADSC graft from green congenic mice. (C, D) GFP-positive (green) PDGFRα^+^Sca-1^+^ ADSC graft was surrounded by recipient astrocytes (labeled with S100β, red). (E, F) Some of GFP-positive (green) PDGFRα^+^Sca-1^+^ ADSCs differentiated into mature neurons (labeled with NEUN, red). Scale bars: 200 μ m in the enlarged panels, 50 μ m in other panels. Nuclei were counterstained with DAPI (C–F, blue). Abbreviations: DAPI, 4′, 6-diamidoino-2-phenylindole.

Because PDGFRα^+^Sca-1^+^ ADSCs could differentiate into neural lineages in vitro, these cells were transplanted into the mouse brain using an in utero transplantation. Transplanted PDGFRα^+^Sca-1^+^ ADSCs were detected in the brain as a mass comprising pleomorphic cells via histology (Figure 2A). Oil red-O staining revealed the presence of intracellular lipid droplets in a clump, suggesting some PDGFRα^+^Sca-1^+^ ADSCs differentiated into mature adipocytes (Figure 2B). In the PDGFRα^+^Sca-1^+^ ADSCs as the green fluorescent protein (GFP) positive population, a small number of the cells expressed a pan-astrocyte marker, S100B, or a mature neuron marker, NEUN (Figure 2C–F). The transplanted clump was surrounded by GFP-negative S100B-positive astrocytes (Figure 2C).

## 4 Maternal ADSC grafts reduce the induction of cytotoxic T cell responses in utero transplantation

**Figure 3:**
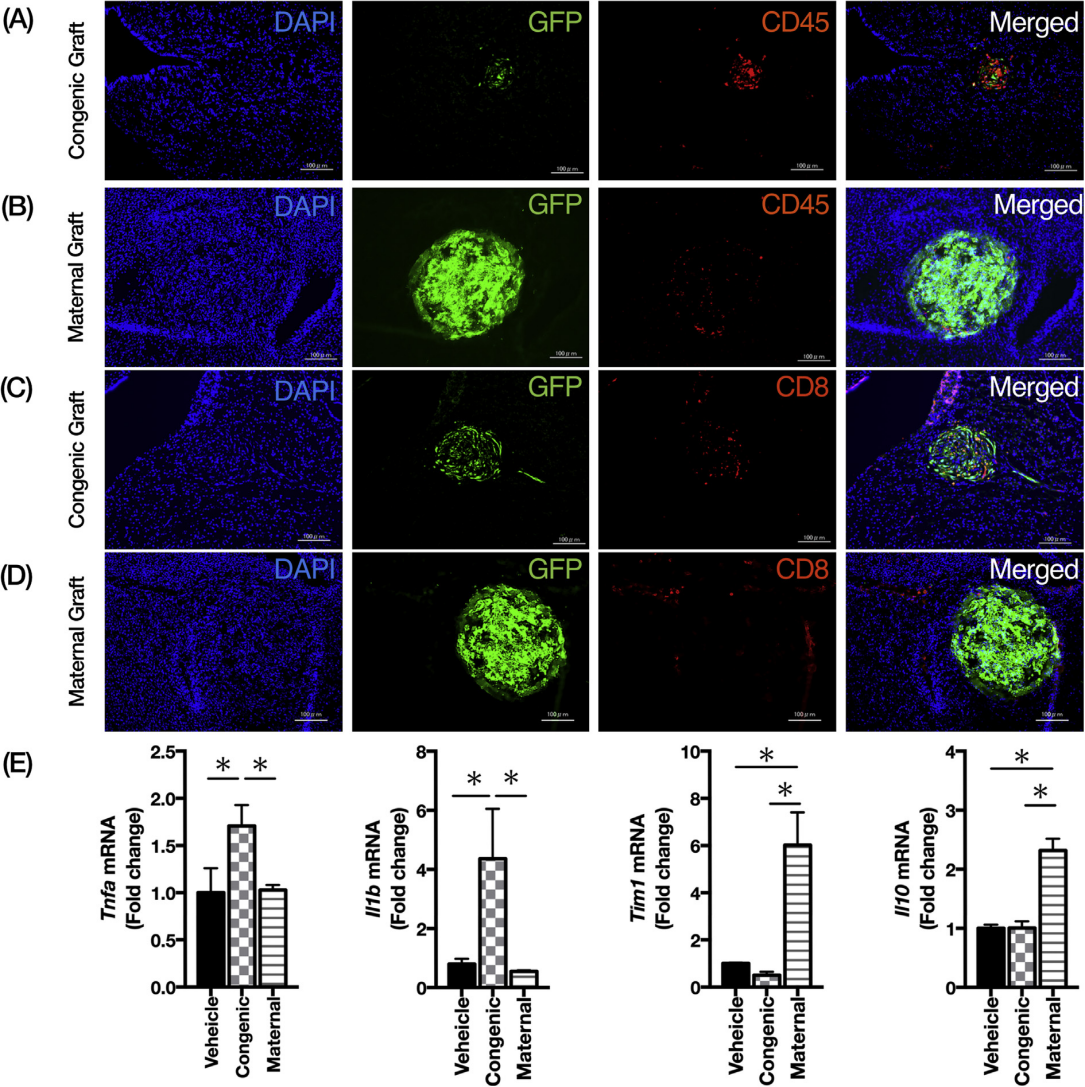
Maternal PDGFRα^+^Sca-1^+^ ADSC grafts reduce the induction of cytotoxic T cell responses in utero transplantation. PDGFRα^+^Sca-1^+^ ADSC from green congenic mice and green fluorescent protein (ZsGreen)-labeled maternal PDGFRα^+^Sca-1^+^ ADSCs were injected into each fetal brain at E14.5 through the uterus. The grafted brains were collected at 5 days after injection and visualized examined using fluorescence. (A) the brain transplanted with PDGFRα^+^Sca-1^+^ ADSCs from green congenic mice were stained with anti-CD45, a pan-leukocyte marker (red). (B) The brain transplanted with Zsgreen-labeled (green) maternal PDGFRα^+^Sca-1^+^ ADSCs were stained with anti-CD45 (red). (C) the brain transplanted with PDGFRα^+^Sca-1^+^ ADSCs from green congenic mice were stained with anti-CD8, a cytotoxic T cell marker (red). (D) The brain transplanted with Zsgreen-labeled (green) maternal PDGFRα^+^Sca-1^+^ ADSCs were stained with anti-CD8 (red). (A–D) Nuclei were counterstained using DAPI (blue). Scale bar; 100μm. (E) RT-qPCR was performed using mRNA isolated from brains with vehicle control (black bar, n = 6), PDGFRα^+^Sca-1^+^ ADSCs from green congenic mice (check pattern bar, n = 6), or maternal PDGFRα^+^Sca-1^+^ ADSCs collected from mice 5 days post-transplantation, to investigate the gene expressions of pro-inflammatory molecules, *Il1b* and *Tnfa*, and anti-inflammatory molecules, *Tim1* and *Il10*. All mRNA expression levels were normalized to the reference gene Actb expression, data presented as fold increase of mean ± SD as the ratios of target genes relative to that of a vehicle in the fetal brain. ∗P < 0.05. Abbreviations: Actb, Actin Beta; DAPI, 4′, 6-diamidoino-2-phenylindole; GFP, green fluorescent protein; qRT-PCR, quantitative reverse transcription-polymerase chain reaction; *Il*, interleukin; *Tnfa*, tumor necrosis factor alfa; *Tim1*, T-cell immunoglobulin and mucin domain 1.

Since PDGFRα^+^Sca-1^+^ ADSCs from green congenic mice were found to elicit a significant adipogenic response against inflammation upon in utero intracranial transplantation, we investigated whether the transplantation with maternal PDGFRα^+^Sca-1^+^ ADSCs could neutralize this induction of inflammation following in utero intracranial transplantation. To this end, we evaluated the occurrence of T cell responses in the transplanted brain at 5 days after in utero intracerebroventricular transplantation with PDGFRα^+^Sca-1^+^ ADSCs from green congenic mice or maternal PDGFRα^+^Sca-1^+^ ADSCs transfected with Zsgreen. The fetuses with maternal ADSCs grafts were characterized by infiltration with small numbers of CD45^+^ cells and CD8^+^ T cells (Figures 3A-D). To further characterize the immune responses to the in utero transplantation with maternal PDGFRα^+^Sca-1^+^ ADSCs graft, we assessed the gene expression of the pro-inflammatory cytokines and the anti-inflammatory cytokines. IL-1β and TNF-α play a critical role in the regulation of inflammation and immune response. The gene expression of the pro-inflammatory cytokines *Il1b* and *Tnfa* levels were found to be significantly increased in the mouse brains with PDGFRα^+^Sca-1^+^ ADSCs from green congenic mice as compared with the vehicle group and the maternal PDGFRα^+^Sca-1^+^ ADSC group (Figure 3E). Intriguingly, the gene expression of the anti-inflammatory cytokines, *Tim1*, and *Il10*, were significantly higher in mouse brains with the maternal PDGFRα^+^Sca-1^+^ ADSC group than those of the other groups (Figure 3E).

## 5 Discussion

Herein, we demonstrated that appropriate mechanical stimulation and three-dimensional culture conditions are conducive to the differentiation of PDGFRα^+^Sca-1^+^ ADSCs into neural lineages. We also revealed that PDGFRα^+^Sca-1^+^ ADSCs assumed neuronal forms, expressed various specific neuronal genes, and survived long-term. Furthermore, we revealed that maternal PDGFRα^+^Sca-1^+^ ADSCs could also reduce the infiltration of cytotoxic lymphocytes. Thus, maternal PDGFRα^+^Sca-1^+^ ADSCs represent an excellent cell source for in utero transplantation because of the potential for neural differentiation and their immune tolerance for the maternal immune system.

Stem cell-based approaches hold great promise for reconstruction and replacement of brain tissue that is lost to injury or neurodegenerative diseases. The potential mechanisms for the therapeutic effect of MSCs may involve the secretion of neurotrophic factors that promote neural cell survival and growth, enhancing neurogenesis, and modulating neuroinflammatory responses [27]. Migration of bone marrow mesenchymal stem cells (BM-MSCs) to the brain and their differentiation into some types of brain cells have been reported in rodents [28] and humans [29]. However, a potential risk related to MSC application might paradoxically arise from the ability of MSCs to suppress immune responses and promote tumor growth and metastasis [30]. These inconsistent results might be related to the heterogeneous nature of the MSC populations, in which the microenvironment probably influences the behavior of the MSCs [31]. The subset PDGFRα^+^Sca-1^+^BM-MSCs partially originate from neural crest cells, and a neural crest origin may better account for the existence of MSCs with neurogenic potential [32, 33]. Our study demonstrated that the aggregates of the subset PDGFRα^+^Sca-1^+^ in ADSCs have differing abilities to differentiate into neuronal lineages. These PDGFRα^+^Sca-1^+^ ADSCs could be useful for MSC transplantation with regard to being less heterogeneous and the ability of neural differentiation.

In our animal models, we found that transplanted PDGFRα^+^Sca-1^+^ ADSCs grafted from green congenic mice survived and grew in the brain expressing markers of neural lineages, including astrocytes and neurons. However, the numbers of the PDGFRα^+^Sca-1^+^ ADSCs expressing the neural lineage markers were small. In the adult animal model, the central nervous system (CNS) resident microglia and macrophages are the first cells to be recruited to sites of CNS transplantation along with neutrophils [34]. Given their capacity for free radical production, phagocytosis, and pro-inflammatory cytokine secretion (i.e., IL-1β and TNF-α), microglia/macrophages can induce direct cell death of transplanted cells, while binding of complement and antibodies to foreign material enables these phagocytes to target allogeneic cells [35]. Our results illustrated that PDGFRα^+^Sca-1^+^ ADSC from green congenic mice induced reactive astrocytes exhibiting S100B, which is constitutively released from reactive astrocytes to confer neuroprotection against oxidative stress at low concentrations and induce inflammation at high concentrations [36]. Reactive astrocytes also secrete pro-inflammatory mediators and neurotoxic factors that amplify neurodegeneration [37]. These reports suggested that the attenuation of astrocyte reactivity following transplantation alters the environment to influence neurogenesis of the transplanted PDGFRα^+^Sca-1^+^ ADSCs.

Our data indicates that the inflammation after transplantation in brain tissue might drive abnormal adipogenesis. It is known that ADSCs are capable of differentiating along multiple pathways, including the adipogenic pathway [17]. Inappropriate adipogenic differentiation of MSCs is associated with several inflammatory disorders because of ectopic fat deposits and alterations in local adipose tissue [38, 39]. Indeed, PDGFRα^+^ skeletal muscle MSC was identified as the source of fat deposits in a murine model of glycerol-induced muscle fiber degeneration [39]. Importantly, changes in the phenotype of MSCs at sites of chronic inflammation may contribute to their inappropriate adipogenesis. Attenuation of adipogenesis may be an essential process during the differentiation of PDGFRα^+^Sca-1^+^ ADSCs into neural cells. The magnitude of inflammation and the neurogenic capacities of ADSCs supports the hypothesis that the inflammatory situation alters MSC characteristics [40]. Therefore, reduction of inflammation after PDGFRα^+^Sca-1^+^ ADSC transplantation may lead transplanted PDGFRα^+^Sca-1^+^ ADSCs to differentiate into neuronal cells without aberrant adipogenic differentiation.

Increased T cell trafficking with fetal intervention has been suggested as a mechanism by which maternal cells respond to the transplanted cells and limit their engraftment after in utero stem cell transplantation. Donor-specific maternal immunoglobulin can reportedly precipitate prenatal donor cell rejection in the fetus [41]. Graft survival in the brain appears to be dependent on the reaction of the innate immune responses organized by microglia and astrocytes at the time of transplantation [42].

CD45 is a common leukocyte antigen expressed by almost all nucleated white cells, including T cells, natural killer cells, and granulocytes [43]. In this study, the reduced number of CD45^+^ cells in mice transplanted with maternal PDGFRα^+^Sca-1^+^ ADSCs, compared to PDGFRα^+^Sca-1^+^ ADSCs from green congenic mice at day 5 post-transplantation. We also observed a reduction in both the innate host immune response with lower numbers of infiltrating CD8^+^ cells at the site of transplantation in the animals transplanted with maternal PDGFRα^+^Sca-1^+^ ADSCs. We showed that maternal PDGFRα^+^Sca-1^+^ ADSC grafts did not increase the gene expressions of the pro-inflammatory molecules compared to PDGFRα^+^Sca-1^+^ ADSC grafts from green congenic mice, whereas maternal PDGFRα^+^Sca-1^+^ ADSC graft increased the gene expressions of TIM-1 and IL-10, which play a critical role in the regulation of the immune response [44]. IL-10 modulates the production by T cells of pro-inflammatory molecules, like TNFα and IL-1 β [34]. These results suggest that maternal PDGFRα^+^Sca-1^+^ ADSC grafts can be used as the donor cells for in utero transplantation to suppress the recipient's immune system, including maternal immunity more than PDGFRα^+^Sca-1^+^ ADSC grafts from green congenic mice. The extent of congenicity between wild-type C57BL/6J and GFP transgenic mice on an inter-substrain hybrid betweenC57/BL6J and C57BL/6CrSlc background are likely to be different, as they were derived and are maintained in different ways including derivation directly from an embryonic stem cell line, backcrossing [45].

The rationale behind in utero transplantation with maternal ADSCs is that maternal stem cells will eventually colonize the fetus's neural stem cell pool of the central nervous system and delay neural metabolic disease progression through tropic support, anti-inflammatory, or intrinsic immune modulatory properties. The benefit is that after the diagnosis of the fetus' disease in the first trimester before constructing fetus' central nervous system using maternal cell-free DNA for prenatal diagnosis, when we can use mother's stem cells for the fetus, we need not spend the time exploring the donner for the appropriateness of transplantation to the fetus. However, if the mothers are not carriers and have the two pathogenic genes, they are not appropriate as the donner and we should not use their cells.

Nevertheless, there were several limitations to this study. First, despite the evidence of immunomodulatory effects exerted by maternal PDGFRα^+^Sca-1^+^ ADSCs, several other factors may have attributed to differentiate neural cells. The second limitation of this study is the lack of data beyond five days post-transplantation with maternal PDGFRα^+^Sca-1^+^ ADSCs grafts.

Thirdly, we transplanted fetal mice with ADSCs from the strain of mice that were matched to MHC class I of the fetus and the mother. We need a further investigation to clear the maternal immune system playing the critical role in triggering in utero transplantation rejection. We should study the effects of transplanting fetal mice with ADSCs from a second strain of mice that were not matched to the fetus of the mother. Moreover, we compared GFP-positive cells among the groups of the fetus transplanted with ADSCs from green congenic mice or ADSCs transfected with Zsgreen gene from mothers. There is a possibility that the difference between green fluorescent proteins affect the immune response after transplantation.

## 6 Conclusion

Our results indicate that maternal PDGFRα^+^Sca-1^+^ ADSCs have the possibility of differentiation into neuronal lineages and suppression of immune response by limiting the inflammation following in utero transplantation. Bearing in mind the uncertainty that PDGFRα^+^Sca-1^+^ ADSCs may or may not be able to restore the functionalities of damaged neural tissue, maternal PDGFRα^+^Sca-1^+^ ADSCs could be used as the donor mesenchymal cell source for in utero transplantation. Further studies are needed to address the additional effects of maternal PDGFRα^+^Sca-1^+^ ADSCs on in utero transplantation, including their effects on the synaptic formation and consequent functional recovery.

## Declarations

### Author contribution statement

Akihiro Kawashima: Conceived and designed the experiments; Performed the experiments; Analyzed and interpreted the data; Contributed reagents, materials, analysis tools or data; Wrote the paper.

Rika Yasuhara: Performed the experiments; Analyzed and interpreted the data.

Ryosuke Akino: Analyzed and interpreted the data.

Kenji Mishima, Michiko Nasu, Akihiko Sekizawa: Contributed reagents, materials, analysis tools or data.

### Funding statement

This work was supported by the Ministry of Education via a Science, Sports and Culture Grant-in-Aid for Young Scientists (B) (Grant Number JP17K16311). The study was partially supported by Ogyaa Foundation.

### Competing interest statement

The authors declare no conflict of interest.

### Additional information

No additional information is available for this paper.

## References

[1] Hennet T. (2012). Diseases of glycosylation beyond classical congenital disorders of glycosylation. Biochim. Biophys. Acta Gen. Subj..

[2] Radke J., Stenzel W., Goebel H.H. (2018). Neurometabolic and Neurodegenerative Diseases in Children, Handbook of Clinical Neurology.

[3] Yin A.H., Peng C.F., Zhao X. (2015). Noninvasive detection of fetal subchromosomal abnormalities by semiconductor sequencing of maternal plasma DNA. Proc. Natl. Acad. Sci. U. S. A..

[4] Yu S.C., Jiang P., Choy K.W. (2013). Noninvasive prenatal molecular karyotyping from maternal plasma. PloS One.

[5] Uys J.D., Reissner K.J. (2011). Glutamatergic Neuroplasticity in Cocaine Addiction, Progress in Molecular Biology and Translational Science.

[6] Trounson A., McDonald C. (2015). Stem cell therapies in clinical trials: progress and challenges. Cell stem cell.

[7] Pearson E.G., Flake A.W. (2013). Stem Cell and Genetic Therapies for the Fetus, Seminars in Pediatric Surgery.

[8] Almeida-Porada G., Atala A., Porada C.D. (2016). In Utero stem cell transplantation and gene therapy: rationale, history, and recent advances toward clinical application. Mol. Ther. Methods Clin. Dev..

[9] McClain L.E., Flake A.W. (2016). In Utero stem cell transplantation and gene therapy: recent progress and the potential for clinical application. Best Pract. Res. Clin. Obstet. Gynaecol..

[10] Steinberg G.K., Kondziolka D., Wechsler L.R. (2016). Clinical outcomes of transplanted modified bone marrow–derived mesenchymal stem cells in stroke: a phase 1/2a study. Stroke.

[11] Kalladka D., Sinden J., Pollock K. (2016). Human neural stem cells in patients with chronic ischaemic stroke (PISCES): a phase 1, first-in-man study. Lancet.

[12] Tfilin M., Sudai E., Merenlender A. (2010). Mesenchymal stem cells increase hippocampal neurogenesis and counteract depressive-like behavior. Mol. Psychiatr..

[13] Ahn S.Y., Chang Y.S., Sung S.I. (2018). Mesenchymal stem cells for severe intraventricular hemorrhage in preterm infants: phase I dose-escalation clinical trial. Stem Cell. Transl. Med..

[14] El Haddad N., Heathcote D., Moore R. (2011). Mesenchymal stem cells express serine protease inhibitor to evade the host immune response. Blood.

[15] Peister A., Mellad J.A., Larson B.L. (2004). Adult stem cells from bone marrow (MSCs) isolated from different strains of inbred mice vary in surface epitopes, rates of proliferation, and differentiation potential. Blood.

[16] Mabuchi Y., Houlihan D.D., Akazawa C. (2013). Prospective isolation of murine and human bone marrow mesenchymal stem cells based on surface markers. Stem Cell. Int..

[17] Zuk P.A., Zhu M., Mizuno H. (2001). Multilineage cells from human adipose tissue: implications for cell-based therapies. Tissue Eng..

[18] Bahmani L., Taha M.F., Javeri A. (2014). Coculture with embryonic stem cells improves neural differentiation of adipose tissue-derived stem cells. Neuroscience.

[19] Kern S., Eichler H., Stoeve J. (2006). Comparative analysis of mesenchymal stem cells from bone marrow, umbilical cord blood, or adipose tissue. Stem cells.

[20] Le Blanc K., Tammik C., Rosendahl K. (2003). HLA expression and immunologic propertiesof differentiated and undifferentiated mesenchymal stem cells. Exp. Hematol..

[21] Ankrum J.A., Ong J.F., Karp J.M. (2014). Mesenchymal stem cells: immune evasive, not immune privileged. Nat. Biotechnol..

[22] Rossignol J., Boyer C., Thinard R. (2009). Mesenchymal stem cells induce a weak immune response in the rat striatum after allo or xenotransplantation. J. Cell Mol. Med..

[23] Griffin M.D., Ryan A.E., Alagesan S. (2013). Anti-donor immune responses elicited by allogeneic mesenchymal stem cells: what have we learned so far?. Immunol. Cell Biol..

[24] Liechty K.W., MacKenzie T.C., Shaaban A.F. (2000). Human mesenchymal stem cells engraft and demonstrate site-specific differentiation after in utero transplantation in sheep. Nat. Med..

[25] Nijagal A., Wegorzewska M., Jarvis E. (2011). Maternal T cells limit engraftment after in utero hematopoietic cell transplantation in mice. J. Clin. Invest..

[26] Delcroix G.J., Curtis K.M., Schiller P.C. (2010). EGF and bFGF pre-treatment enhances neural specification and the response to neuronal commitment of MIAMI cells. Differentiation.

[27] Volkman R., Offen D. (2017). Concise review: mesenchymal stem cells in neurodegenerative diseases. Stem cell..

[28] Mezey E., Chandross K.J., Harta G. (2000). Turning blood into brain: cells bearing neuronal antigens generated in vivo from bone marrow. Science.

[29] Cogle C.R., Yachnis A.T., Laywell E.D. (2004). Bone marrow transdifferentiation in brain after transplantation: a retrospective study. Lancet.

[30] Uccelli A., Moretta L., Pistoia V. (2008). Mesenchymal stem cells in health and disease. Nat. Rev. Immunol..

[31] Liotta L.A., Kohn E.C. (2001). The microenvironment of the tumour–host interface. Nature.

[32] Morikawa S., Mabuchi Y., Niibe K. (2009). Development of mesenchymal stem cells partially originate from the neural crest. Biochem. Biophys. Res. Commun..

[33] Morikawa S., Mabuchi Y., Kubota Y. (2009). Prospective identification, isolation, and systemic transplantation of multipotent mesenchymal stem cells in murine bone marrow. J. Exp. Med..

[34] Hoornaert C.J., Luyckx E., Reekmans K. (2016). In Vivo interleukin-13-primed macrophages contribute to reduced alloantigen-specific T cell activation and prolong immunological survival of allogeneic mesenchymal stem cell implants. Stem Cell..

[35] Wasowska B.A., Lee C.-Y., Halushka M.K. (2007). New concepts of complement in allorecognition and graft rejection. Cell. Immunol..

[36] Donato R. (2003). Intracellular and extracellular roles of S100 proteins. Microsc. Res. Tech..

[37] Zilka N., Kazmerova Z., Jadhav S. (2012). Who fans the flames of Alzheimer's disease brains? Misfolded tau on the crossroad of neurodegenerative and inflammatory pathways. J. Neuroinflammation.

[38] Goldfarb J.W., Roth M., Han J. (2009). Myocardial fat deposition after left ventricular myocardial infarction: assessment by using MR water-fat separation imaging. Radiology.

[39] Uezumi A., Fukada S.-i., Yamamoto N. (2010). Mesenchymal progenitors distinct from satellite cells contribute to ectopic fat cell formation in skeletal muscle. Nat. Cell Biol..

[40] Jones E., Churchman S., English A. (2010). Mesenchymal stem cells in rheumatoid synovium: enumeration and functional assessment in relation to synovial inflammation level. Ann. Rheum. Dis..

[41] Riley J.S., McClain L.E., Stratigis J.D. (2018). Pre-existing maternal antibodies cause rapid prenatal rejection of allotransplants in the mouse model of in utero hematopoietic cell transplantation. J. Immunol..

[42] Reekmans K., Praet J., Daans J. (2012). Current challenges for the advancement of neural stem cell biology and transplantation research. Stem Cell Rev..

[43] Penninger J.M., Irie-Sasaki J., Sasaki T. (2001). CD45: new jobs for an old acquaintance. Nat. Immunol..

[44] Ding Q., Yeung M., Camirand G. (2011). Regulatory B cells are identified by expression of TIM-1 and can be induced through TIM-1 ligation to promote tolerance in mice. J. Clin. Invest..

[45] Kallenbach L.R., Bianchi D.W., Peter I. (2011). Maternal background strain influences fetal–maternal trafficking more than maternal immune competence in mice. J. Reprod. Immunol..

